# Transient splenial lesion: Further experience with two cases

**DOI:** 10.4103/0971-3026.73531

**Published:** 2010-11

**Authors:** Paramjeet Singh, Dhrubajyoti Gogoi, Sameer Vyas, Niranjan Khandelwal

**Affiliations:** Department of Radiodiagnosis, Postgraduate Institute of Medical Education and Research, Chandigarh, India

**Keywords:** MRI, splenium of corpus callosum, transient splenium lesion

## Abstract

Transient splenial lesions (TSL) of the corpus callosum are uncommon radiologic findings that are seen in a number of clinical conditions with varied etiologies. They were first described a decade earlier in patients with epilepsy and hence were thought to be seizure or seizure therapy related. Subsequently, more cases were described by different observers in diseases with different etiologies, and the list is still increasing. Awareness of these lesions is necessary as they are an uncommon finding and have to be differentiated from other infective/noninfective causes. MRI is the imaging modality of choice as these lesions are not seen on routine noncontrast CT scan. The authors here describe two cases which showed TSL, with complete/partial resolution on follow-up scans. The authors also present a review of the literature.

## Introduction

Transient splenial lesions (TSL) are rare radiologic findings that may be seen in a wide spectrum of diseases.[1–13] These reversible lesions are oval homogenous, non-hemorrhagic, and non-enhancing lesions seen in the central part of the splenium of the corpus callosum (SCC). The lesions are bright on T2W and FLAIR images; they are slightly hypointense on T1W images and show restricted diffusion.[1–4] These lesions are usually incidentally detected when imaging is done for encephalopathy/encephalitis or seizures, and the actual incidence might be more than what reports indicate.[3] Recognizing these lesions is important, both for the treating physician as well as the radiologist, as the differential diagnoses include a wide spectrum of conditions with varying clinical significance.

## Case Reports

### Case 1

**Figure 1 (A–D):**
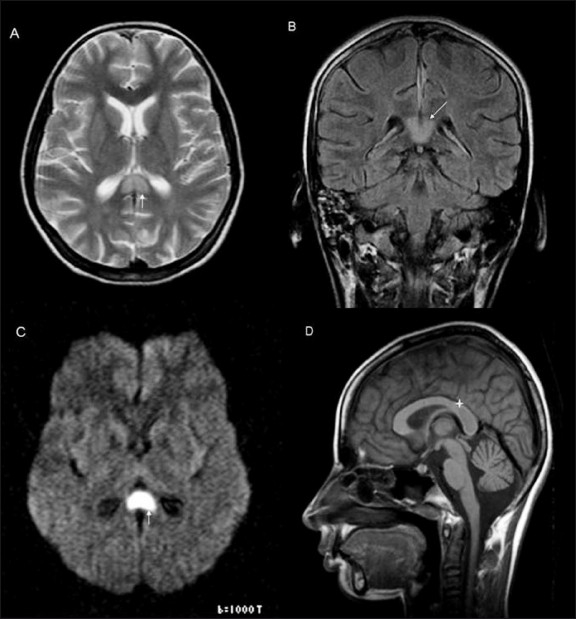
Axial T2W (A), coronal FLAIR (B), and diffusionweighted (C) images show a hyperintense, well-defined lesion (white arrow) in the splenium of the corpus callosum with a thin rim of surrounding non-hyperintense white matter. The same lesion on the sagittal T1W image (D) shows hypo to isointense signal (star)

**Figure 2 (A–C):**
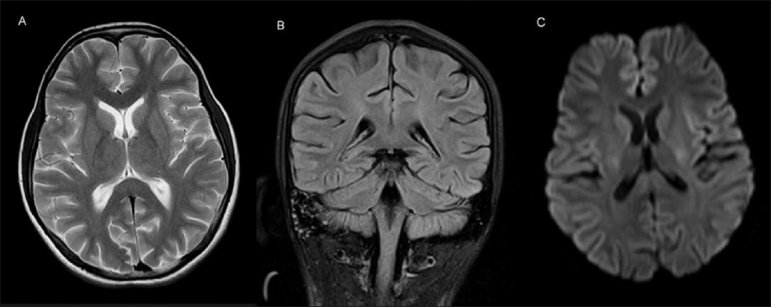
Axial T2W (A), coronal FLAIR (B), and diffusion-weighted (C) images show complete disappearance of the lesion seen in Figure 1

A 17-year-old female with chronic renal parenchymal disease and congenital blindness presented with altered behavior for one day. She complained of generalized weakness, loss of appetite, and easy fatigability for 15 days. There was no history of fever or loss of consciousness or seizures. General physical and systemic examination was normal. Laboratory examination showed decreased hemoglobin (6.5 mg/dl) and increased blood urea (193 mg%) and serum creatinine (9.3 mg%). USG of the abdomen showed small echogenic kidneys. MRI revealed a well-defined hyperintense lesion on T2W imaging and FLAIR in the SCC [Figure 1] with restricted diffusion. A follow-up MRI performed eight days later showed complete disappearance of the lesion with restoration of normal diffusion [Figure 2]. There was clinical improvement and the patient was discharged.

### Case 2

**Figure 3 (A–C):**
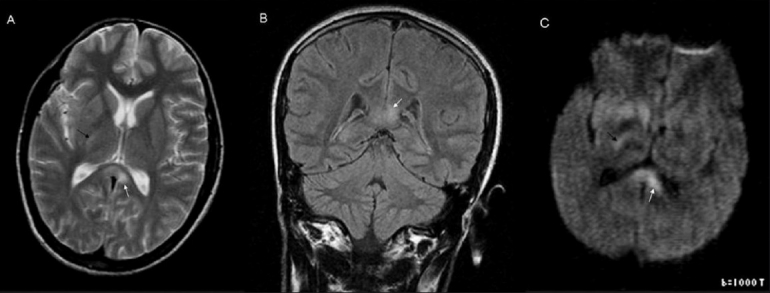
Axial T2W (A), coronal FLAIR (B), and diffusion-weighted (C) images show a hyperintense lesion (white arrow) in the splenium of the corpus callosum, left of the midline. Note the ill-defined margin with the surrounding white matter. The axial T2W (A) and diffusion-weighted (C) images also show ill-defined hyperintensity in the posterior limb of the right internal capsule (black arrow)

**Figure 4 (A–C):**
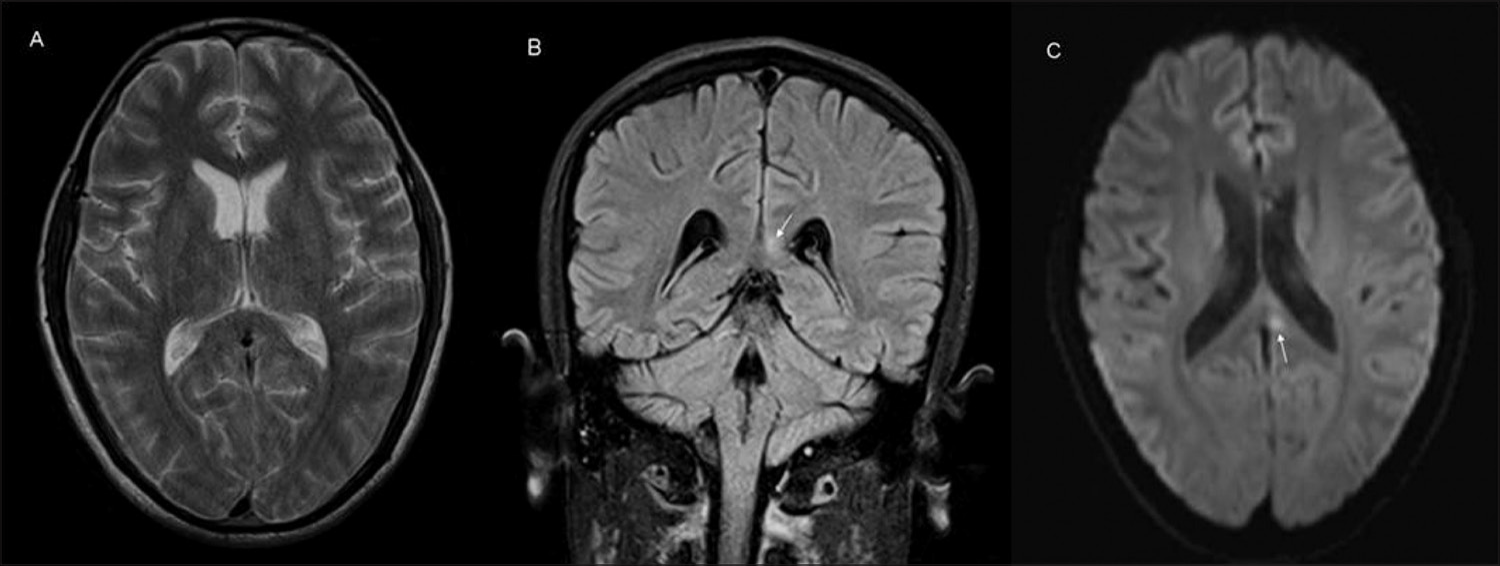
Axial T2W (A), coronal FLAIR (B), and diffusion-weighted (C) images show mild persistent hyperintensity (white arrow) in the corpus callosum, which however is less than in Figure 3

An 18-year-old male presented with altered sensorium, violent behavior, and confusion. There was no preceding history of fever, seizures, trauma, or any addiction. On examination, there was left-sided hemiparesis and urinary and bowel incontinence. General physical and systemic examination was normal. There were no meningeal signs. Laboratory tests including hemogram, renal function test, serum electrolytes, and cerebrospinal fluid (CSF) were within normal limits. EEG showed diffuse encephalopathy. The workup for Japanese encephalitis (IgM antibody) was negative. MRI showed an ovoid hyperintense lesion with ill-defined margins on T2W imaging and FLAIR; the lesion was more towards the left of the midline in the SCC [Figure 3]. The lesion also showed evidence of restricted diffusion on diffusion-weighted imaging (DWI) and in the apparent diffusion coefficient (ADC) maps. Follow-up MRI on day 15 showed significantly decreased signal intensity, with mild persistent T2 hyperintensity, especially on the left side of midline in the SCC [Figure 4]. In comparison to the previous scan the lesion had substantially decreased in size and the patient had recovered clinically.

## Discussion

MRI findings in both our cases were consistent with the diagnosis of TSL: the lesions were seen in the classical location and were isolated findings that eventually disappeared. The clinical outcome was good in both cases.

Transient splenial lesions (TSL) are seen in conditions with varied etiologies, including epilepsy, the usage as well as sudden withdrawal of antiepileptic drugs (AED), brain infarction, multiple sclerosis; cerebral trauma, neoplasm, adrenoleukodystrophy, AIDS dementia complex,[2,5–7] infections like influenza, measles, herpes, salmonella, mumps, adenovirus, varicella zoster, Legionnaires disease, rotavirus,[5] HIV, tubercular meningitis[3] and other conditions like hypoglycemia’[13] Marchiafava–Bignami syndrome, and hemolytic-uremic syndrome with encephalopathy.[8] Mild encephalitis/encephalopathy with a reversible isolated SCC lesion (MERS) is a recently described clinicoradiological syndrome with excellent prognosis.[9] Encephalopathy/encephalitis can be differentiated from the rest of the conditions by the clinical course and laboratory findings.[9] Where an infective etiology or encephalitis can be ruled out, the term encephalopathy is used;[5,9,10] examples include Reye syndrome and influenza-associated encephalitis/encephalopathy (IAEE).

TSL was first described in epilepsy by Chason *et al*. in 1996 and Kim *et al*. in 1999. The exact mechanism of the development of these lesions is not known. Some authors have implicated a transient breakdown of the blood–brain barrier due to focal edema of the splenium in the postictal period, as the SCC contains decussating fibers originating from the temporal lobes which may be involved in secondary generalization.[1,11] Kim *et al*. did not agree with this theory and considered the lesions to be due to possible AED toxicity-induced reversible demyelination. Other postulated mechanisms are reversible extrapontine osmotic myelinolysis due to sodium and glucose imbalance and due to toxicity or hypersensitivity to AEDs or upon their withdrawal.[2,3,12] TSL is also seen in the hemolytic-uremic syndrome with encephalopathy, where the possible mechanism is transient local edema caused by *Escherichia coli* verotoxin-induced microvascular angiopathy.[8]

These lesions can be differentiated from those of multiple sclerosis, acute disseminated encephalomyelitis (ADEM), and encephalitis, as the lesions in these latter conditions are asymmetric and have irregular inflammatory margins.[3] ADEM is characterized by multiple subcortical T2-hyperintense lesions that are nearly always asymmetric. The corpus callosum, whenever involved, also shows this asymmetry.[7,10] The white matter damage can be permanent. Some studies have tried to characterize these lesions by their MRI appearances and to age them as either early or late lesions. A well-defined ovoid lesion forming an acute angle with the SCC and which is mildly swollen and surrounded by a rim of normal myelin is labeled as an early lesion, while those with loss of clear demarcation from the surroundings are labeled as late lesions.[3]

The spectrum of conditions with TSL has been recently expanded to include MERS and IAEE.[5–7,10] In MERS, in addition to the SCC lesions, bilateral parietal and frontoparietal lesions are also observed. The SCC lesions also spread into the lateral portions of the corpus callosum.[10] Among the proposed mechanisms for TSL in IAEE, the widely accepted one is the theory of intramyelinic edema due to inflammation and subsequent migration of inflammatory cells, with associated cytotoxic edema.[5,9] The other mechanisms suggested for the development of SCC include a cytokine-mediated immunologic reaction leading to microvascular endothelial injury and perivascular edema,[5] and direct viral invasion of neurons.[5]

In our patients, any of the above mechanisms may have been responsible. In the first patient, the associated renal parenchymal disease may have been the culprit causing osmotic imbalance leading to TSL. In this case both the theories regarding osmotic demyelination and intramyelinic edema may explain the findings. This is supported by the fact that the lesions totally disappeared without a trace after eight days.

In the second case, mild encephalitis/encephalopathy (MERS) may be the underlying factor, leading to either intramyelinic edema or inflammatory exudates.[10,14] The lesion in this patient was irregular and asymmetric, with poor distinction between the TSL and peripheral normal myelin. There was asymmetric lateral extension [Figures 3 and 4] and additional lesions could also be seen involving the right internal capsule, explaining his left hemiparesis. The lesion had not completely resolved after 15 days of follow-up. However, it is possible that the lesion may be in a state of partial resolution as previous reports have documented regression over longer periods of follow-up (up to 3 months).

In both our cases there was no history of seizures or treatment with AED. The laboratory and MRI findings did not support a diagnosis of encephalitis or ADEM. We could also rule out other potential causes of TSL in these two patients.

To conclude, whenever this finding is noted it should immediately arouse suspicion of the aforementioned possibilities, which can lead to appropriate clinical decision-making and patient management. TSL is an isolated, silent, reversible lesion and should not be mistaken for something more sinister.
